# Action Mode of Gut Motility, Fluid and Electrolyte Transport in Chronic Constipation

**DOI:** 10.3389/fphar.2021.630249

**Published:** 2021-07-27

**Authors:** Qi Zhao, Yan-Yan Chen, Ding-Qiao Xu, Shi-Jun Yue, Rui-Jia Fu, Jie Yang, Li-Ming Xing, Yu-Ping Tang

**Affiliations:** Key Laboratory of Shaanxi Administration of Traditional Chinese Medicine for TCM Compatibility, and State Key Laboratory of Research and Development of Characteristic Qin Medicine Resources (Cultivation), and Shaanxi Key Laboratory of Chinese Medicine Fundamentals and New Drugs Research, Shaanxi University of Chinese Medicine, Xi’an, China

**Keywords:** pharmacological treatment, pathophysiology, gut motility, fluid and electrolyte transport, chronic constipation

## Abstract

Chronic constipation is a common gastrointestinal disorder, with a worldwide incidence of 14–30%. It negatively affects quality of life and is associated with a considerable economic burden. As a disease with multiple etiologies and risk factors, it is important to understand the pathophysiology of chronic constipation. The purpose of this review is to discuss latest findings on the roles of gut motility, fluid, and electrolyte transport that contribute to chronic constipation, and the main drugs available for treating patients. We conducted searches on PubMed and Google Scholar up to 9 February 2021. MeSH keywords “constipation”, “gastrointestinal motility”, “peristalsis”, “electrolytes”, “fluid”, “aquaporins”, and “medicine” were included. The reference lists of searched articles were reviewed to identify further eligible articles. Studies focusing on opioid-induced constipation, evaluation, and clinic management of constipation were excluded. The occurrence of constipation is inherently connected to disorders of gut motility as well as fluid and electrolyte transport, which involve the nervous system, endocrine signaling, the gastrointestinal microbiota, ion channels, and aquaporins. The mechanisms of action and application of the main drugs are summarized; a better understanding of ion channels and aquaporins may be helpful for new drug development. This review aims to provide a scientific basis that can guide future research on the etiology and treatment of constipation.

## Introduction

Chronic constipation is a common gastrointestinal condition that is characterized by a high incidence rate, complex etiology, and difficult treatment. It can manifest as abdominal pain or bloating, or more commonly as chronic symptoms such as difficulty passing stools, infrequent stools, or incomplete defecation. Sometimes it can last for weeks, months, or years (Lacy et al., 2016). The prevalence of chronic constipation is increasing due to changes in diet composition, accelerated pace of life, and the influence of complex social and psychological factors. Epidemiological surveys have shown that the incidence of chronic constipation is between 14 and 30% worldwide, affecting individuals of all ages, races, socioeconomic status, and nationalities (Camilleri et al., 2017). However, it is perceived as being less frequent and not serious, and is often overlooked. Chronic constipation negatively impacts on quality of life, causing abdominal pain, bloating, loss of appetite and/or nausea, headaches, bad breath, restlessness, anxiety and/or depression, and is associated with a substantial economic burden to individuals and society (Peery et al., 2019). The annual healthcare cost for patients with chronic constipation was reported to be $11,991 in the United States. The direct costs are substantial and include outpatient services (44.8%), inpatient hospitalizations (33.9%), prescriptions (17.8%), and emergency department visits (3.5%) (Cai et al., 2014). The economic burden of chronic constipation includes the costs of medical treatment or hospitalization, as well as those associated with treatment failure, which increase both healthcare resource utilization and medical costs (Guerin et al., 2014).

The mechanisms, evaluation, and management of chronic constipation were recently reviewed. The main mechanisms discussed were colonic sensorimotor dysfunction and alteration of the microbiome (Bharucha and Lacy, 2020). However, the pathogenesis of chronic constipation is multifactorial and includes colonic motility and fluid transport, anorectal movement and sensory functions, as well as dietary and behavioral factors. The purpose of this review is to introduce the latest findings on the role of gut motility and fluid and electrolyte absorption and secretion in the development of chronic constipation.

**FIGURE 1:**
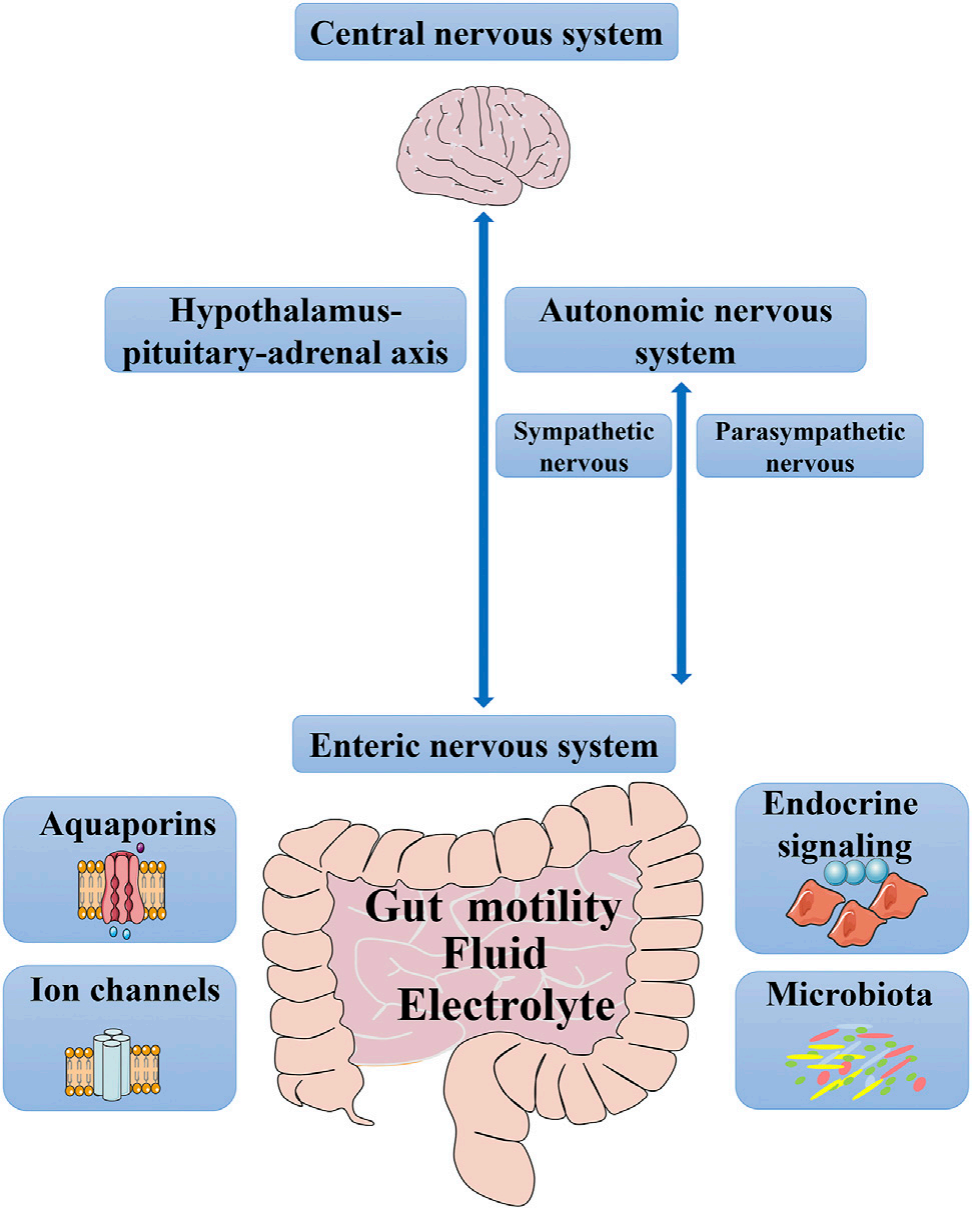
Factors that control gut motility, fluid and electrolyte. From top to bottom, it encompasses the central nervous system, the sympathetic and parasympathetic branches of the autonomic nervous system, hypothalamus-pituitary-adrenal axis, the enteric nervous system, endocrine signaling, microbiota, aquaporins, and ion channels.

Chronic constipation is the earliest manifestation of gastrointestinal hypomotility, which is characterized by significantly prolonged intestinal transit time (Wang, 2015). Slower colonic transport prolongs the retention of intestinal contents and increases the reabsorption of water and electrolytes, resulting in a reduced volume and hardening of stools. Several factors jointly regulate gut motility, fluid, and electrolytes: the enteric nervous system (ENS), autonomic nervous system (ANS), central nervous system (CNS), endocrine signaling, microbiota, ion channels, and aquaporins (AQPs) (Figure 1). An imbalance or dysfunction in any of these components may cause abnormal intestinal function, which can lead to symptoms of constipation. First, gut motility and its influencing factors have been summarized, and then water and electrolyte transport will be discussed, focusing on the complexity of AQPs and ion channels. In terms of intestinal movement, one mechanism is neural networks, which control ENS independently, and the other involves the ANS and CNS. When it comes to fluid and electrolyte, we have mainly focused on channels, such as ion channels and AQPs. Finally, medication for treating chronic constipation has been discussed. Increasing motility and water secretion are currently the main goals of treatment for constipation; therefore, laxatives, which affect gut motility and water absorption/secretion, will be highlighted. Understanding the regulatory mechanism of gut motility and fluid and electrolytes transport in chronic constipation is helpful for further pathophysiological exploration and to provide a necessary theoretical basis for the further use of safe and effective drugs.

## Gut Motility Pattern

In most cases, chronic constipation is considered to be closely related to disorders of gut motility (Wang, 2015). Two main types of intestinal movement enable feces to enter the rectum: peristalsis and colonic propulsion. These movements ensure the regular contraction of the colon wall and enable the contents to advance, thus promoting the normal excretion of feces.

### Peristalsis

**FIGURE 2:**
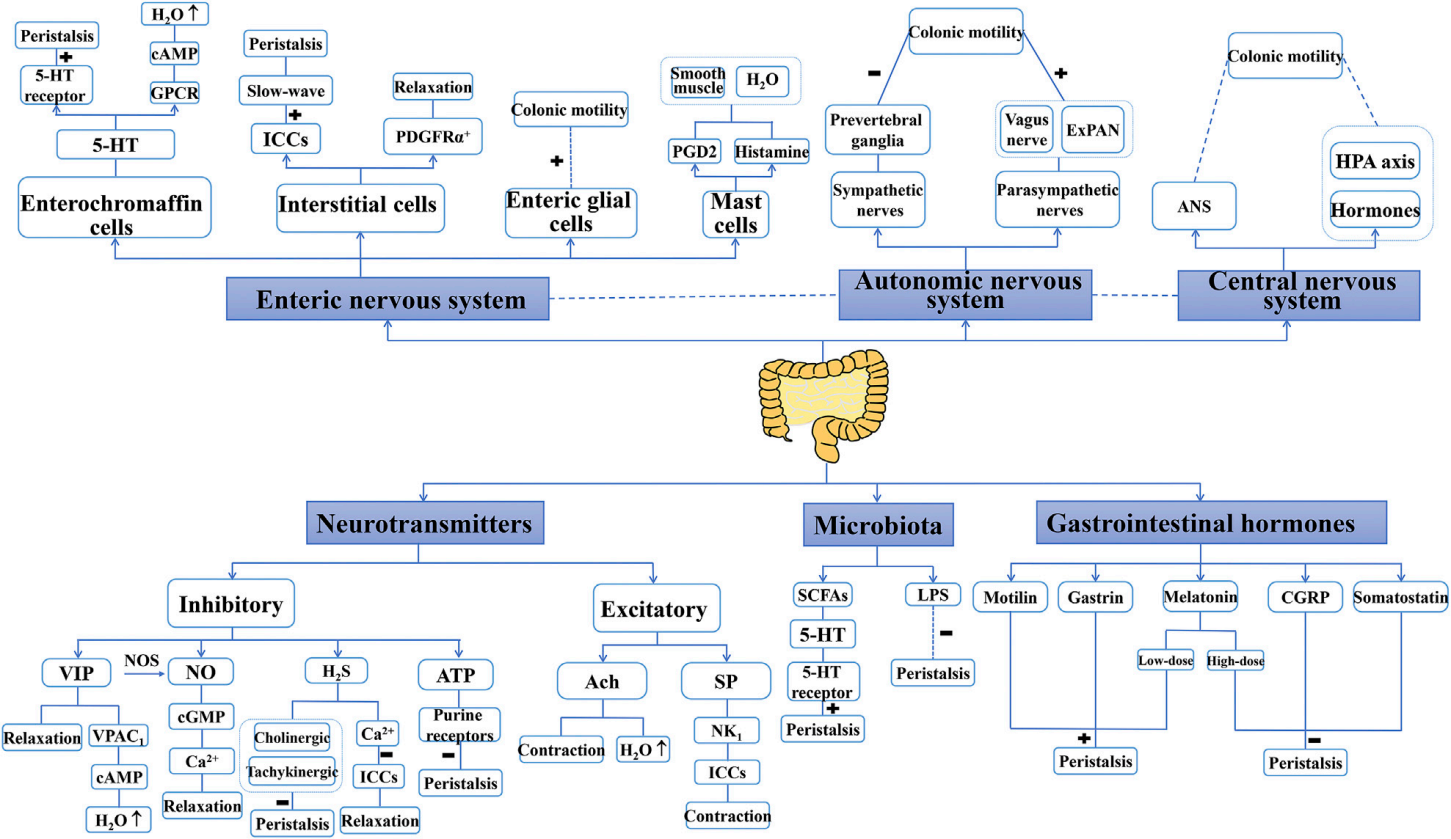
Factors that modulate peristalsis. “+” indicate a promoting role, “−” indicate an inhibition role. Enteric nervous system, autonomic nervous system, central nervous system, neurotransmitters, gastrointestinal hormones and microbiota are the main factors to control peristalsis. Some of these factors also involve the secretion of water. 5-HT: 5-hydroxytryptamine; Ach, acetylcholine; ANS, autonomic nervous system; ATP, adenosine triphosphate; cAMP, cyclic adenosine monophosphate; cGMP, cyclic guanosine monophosphate; CGRP, calcitonin gene-related peptide; ExPANs, extrinsic primary afferent neurons; GPCR, G-protein coupled receptors; HAP axis, hypothalamus–pituitary–adrenal axis; H_2_S, hydrogen sulfide; ICCs, interstitial cells of Cajal; LPS, lipopolysaccharide; NK_1_, neurokinin 1; NO, nitric oxide; NOs, nitric oxide synthase; PDGFRα^+^, platelet-derived growth factor receptor α-positive; PGD2, prostaglandin D2; SCFAs, short-chain fatty acids; SP, substance P; VIP, vasoactive intestinal peptide.

Peristalsis is the basis of most gastrointestinal propulsion movements, mainly in the colon, and includes the coordinated contraction and relaxation of the intestinal muscle layer (Camilleri et al., 2017). Relaxation/inhibition and contraction/excitement are thought to spread along the intestinal tract following stimulation. The neural control hierarchy for peristalsis is as follow: the primary regulator is the ENS, followed by the ANS and then the CNS. Additionally, neurotransmitters, gastrointestinal hormones, and microbiota work together to modulate peristalsis. Factors that modulate peristalsis are shown in Figure 2.

#### Regulation by ENS

The exertion of intestinal motor function is largely achieved by regulation of the ENS, which acts independently from the CNS and is a highly autonomous gastrointestinal neural network mainly composed of enteric neurons and enteric glial cells (EGCs) (Avetisyan et al., 2015). Enteric neurons include afferent neurons, interneurons, motor neurons, mechanosensitive neurons, etc., which are connected in peristalsis reflex circuitries (De et al., 2004). They extend process to communicate with diverse cells types including other enteric neurons, enterochromaffin cells (ECs), interstitial cells, and mast cells (Furness, 2000). EGCs are distributed inside and outside the nerve plexus (between the submucosal nerve plexus and the lamina propria), which can interact with intestinal neurons or directly participate in the regulation of gut motility (Aubé et al., 2006). EGCs help to promote the normal regulation of intestinal electric ion transport and secretory motor function (Grubišić and Gulbransen, 2017).

Peristalsis can be activated by chemical and/or mechanical stimuli that are sensed by ECs (Gershon and Tack, 2007). Most 5-hydroxytryptamine (5-HT) in the human body is secreted by ECs. 5-HT is a mediator involved in regulating a variety of physiological functions in the gastrointestinal tract and plays an important role in regulating gut motility and intestinal secretion (Reynaud et al., 2016). 5-HT can activate the peristaltic reflex by activating the 5-HT receptor on the mucosal end of the intrinsic primary afferent neurons, including 5-HT_2B_, 5-HT_3_, 5-HT_4_ and 5-HT_7_ receptors (Wouters et al., 2007; Smith et al., 2014). These receptors signals through the myenteric plexus, which induces a wave of intestinal smooth muscle contraction. And the propulsion requires activation of both ascending excitatory neurons and descending inhibitory neurons. 5-HT also binds to G-protein coupled receptors in enterocytes (except for 5-HT_3_ receptors), increasing the levels of cyclic adenosine monophosphate (cAMP) in target cells (Smith et al., 2014). An increase in cAMP causes the cystic fibrosis transmembrane conductance regulator (CFTR) to open, leading to Cl^−^ and water outflow (Dawson, 1991). Accordingly, the regulation of 5-HT can have profound effects on gut motility and intestinal secretion.

Interstitial cells produce and transmit electrical signals that regulate the excitement and inhibition of smooth muscle. Two types of interstitial cells are involved: interstitial cells of Cajal (ICCs) and platelet-derived growth factor receptor α positive (PDGFRα^+^) cells. Constipation may be related to changes in the structure and shape of ICCs (Cohen et al., 2017), which play a significant physiological role in gut motility. They form a network of cells around the myenteric plexus between the circular and longitudinal muscle layers of the entire intestine and are responsible for regulating the contraction of gastrointestinal smooth muscle (Komuro, 2006). The pacing activity induced by ICCs causes a rhythmic slow-wave of smooth muscle cells. Slow-wave is a relatively regular periodic electrical activity, which controls the rhythm of intestinal contraction. ICCs integrate slow-wave activity with excitatory and inhibitory neurotransmission to orchestrate peristalsis. These findings demonstrated that disorders of gut motility are due to the loss of slow-wave activity as well as disturbed neurotransmission (Klein et al., 2013). PDGFRα^+^ cells inhibit smooth muscle activity by activating purinergic receptors (Camilleri et al., 2017).

There are close apposed between axons and mast cells in the gastrointestinal mucosa (Stead et al., 1989). Mast cells can regulate peristalsis and fluid secretion *via* bidirectional brain-gut interactions between the ENS and the CNS (Schaeffer et al., 2012). Mast cells can stimulate excitatory neurons and activate the ENS network, and thereby promote powerful propulsive motility (Wang et al., 2014). Histamine and prostaglandin D2 released by mast cells have been shown to modulate the smooth muscle and fluid secretion (Wouters et al., 2016).

#### Regulation by ANS and CNS

The ANS, which includes the sympathetic and parasympathetic nervous systems, is a collection of afferent and efferent neurons that link the CNS with the ENS (Tait and Sayuk, 2021). Both sympathetic and parasympathetic nerves regulate gut motility by affecting the ENS circuit (Mayer et al., 2014). The postganglionic fibers of the sympathetic nerve are adrenergic nerve fibers which cause the release of norepinephrine that elicit the presynaptic inhibition of neurotransmitter release, thereby inhibiting intestinal motility. Meanwhile, the parasympathetic nerve mainly transmits excitation signals *via* the vagus nerve (Bonaz et al., 2018). Extrinsic primary afferent neurons (ExPANs) derived from spinal ganglia can regulate colon function. ExPANs regulate myenteric neuron activity and smooth muscle contraction *via* a parasympathetic spinal circuit (Smith-Edwards et al., 2019).

CNS modulates gastrointestinal motility *via* two distinct routes (Mayer and Tillisch, 2011). First, it modulates gut motility *via* the ANS. The vagus nerve is the main component of the parasympathetic nervous system, which can transmit intestinal-related signals to the CNS. Second, CNS signals induce gastrointestinal motility *via* hormonal pathways, including the hypothalamus–pituitary–adrenal axis, and hormones of the neuroendocrine stress response. For example, neurotensin released by central preganglionic neurons promotes the release of substance P (SP), which in turn stimulates peristalsis (Szurszewski et al., 2002).

#### Regulation by Neurotransmitters

Constipation is associated with abnormalities in intestinal neurotransmitters, which are a class of active small-molecule peptides produced in gastrointestinal endocrine cells and nerve cells. There are two type of neurotransmitters, inhibitory and excitatory ones, which play an important role in regulating the motility and secretion of the gastrointestinal tract (Schneider et al., 2019).

Inhibitory neurotransmitters include vasoactive intestinal peptide (VIP), nitric oxide (NO), adenosine triphosphate (ATP), and hydrogen sulfide (H_2_S), which can induce smooth muscle relaxation and inhibit intestinal sensitivity. VIP, one of the most important neurotransmitters in the ENS, inhibits the contraction of intestinal circular muscle, maintaining the intestine in a state of relaxation, and may also participate in the secretion of intestinal fluid (Mourad and Nassar, 2000). VIP binds to its receptor (VPAC_1_), leading to the excretion of cAMP-related HCO_3_^−^. This causes Na^+^ and H_2_O to enter the intestinal cavity, thus increasing fluid secretion (Chandrasekharan et al., 2013). Therefore, reduced VIP production may result in reduced fluid secretion, which is a possible cause of constipation or its aggravation. VIP can activate nitric oxide synthase (NOS) in the colon wall, leading to the production of the inhibitory neurotransmitter NO. NO activates cyclic guanosine monophosphate (cGMP)-dependent protein kinase by stimulating intracellular soluble guanylate cyclase, which reduces the level of intracellular Ca^2+^, relaxes smooth muscle cells, and eventually weakens gastrointestinal motility (Beck et al., 2019). H_2_S is a gasotransmitter, which plays a role in the regulation of gut motility. In addition, it can inhibit the pacemaker activity of ICCs by regulating intracellular Ca^2+^, which in turn leads to relaxation of gastrointestinal smooth muscle (Parajuli et al., 2010). Furthermore, high concentrations of H_2_S can inhibit gut motility by interacting with cholinergic and tachykinergic neural-mediated pathways (Martinez-Cutillas et al., 2015). ATP, an important neurotransmitter, inhibits peristalsis by acting on the purine receptors on intestinal nerves and muscles (Heinemann et al., 1999).

5-HT, acetylcholine (Ach), and SP are excitatory neurotransmitters, which can stimulate intestinal muscle contraction and promote intestinal peristalsis. 5-HT is a paracrine signaling molecule released from ECs, and also a neurotransmitter that produced by serotonergic neurons of the ENS (Gershon, 2012). Ach activates the gastrointestinal tract by stimulating muscle contraction and increasing the peristalsis of gastrointestinal smooth muscle. Additionally, the non-neuronal release of Ach from colonocytes, coupled with propionate stimulation, promote Cl^−^ secretion, *via* the paracrine action of Ach on muscarinic receptors of colonocytes (Yajima et al., 2011). SP can regulate ICCs through the tachykinin NK_1_ receptor and exerts a strong contractile effect on gastrointestinal smooth muscle (Jun et al., 2004).

#### Regulation by Gastrointestinal Hormones

The ENS regulates gut motility through neurotransmitters and by the secretion of gastrointestinal hormones for humoral regulation. Motilin, gastrin, melatonin, calcitonin gene-related peptide (CGRP), and somatostatin have important physiological significance in the regulation of gastrointestinal motility (Penning et al., 2000). Motilin acts directly on the motilin receptor on gastrointestinal smooth muscle cells and stimulates gastrointestinal peristalsis (Xu et al., 2005). Gastrin can increase small intestinal motility (Ahmed and Ahmed, 2019). Melatonin acts on the muscularis mucosae or the myenteric plexus, is involved in regulating colonic motility, and has a bidirectional effect on gut motility (Esteban-Zubero et al., 2017). Low-dose melatonin has been shown to accelerate intestinal transit, while high-dose melatonin decreases gut motility. CGRP has been found to exert an inhibitory effect on gut motility by inducing interneurons to trigger the peristaltic reflex (Ceccotti et al., 2018). Somatostatin inhibits intestinal secretion, peristalsis, and the release of gastrointestinal hormones by acting on the somatostatin receptors on gastric smooth muscle (John and Chokhavatia, 2017).

#### Regulation by Microbiota

Studies have revealed an important relationship between the intestinal microbiota and constipation (Yarullina et al., 2020). *Bacteroides* were found to be more abundant in patients with chronic constipation (Yarullina et al., 2020), as well as decreased numbers of bifidobacteria and lactobacilli, compared with healthy controls (Dimidi et al., 2017). Short-chain fatty acids (SCFAs), endotoxin, and other products are produced during intestinal microbial metabolism and may affect gut motility (Segers et al., 2019). The intestinal microbiota can regulate the release of 5-HT by ECs, which in turn, affects gut motility (Yano et al., 2015). Furthermore, the release of 5-HT from ECs in response to SCFAs stimulates 5-HT_3_ receptors located on vagal sensory fibers, resulting in muscle contraction (Fukumoto et al., 2003). Serum endotoxin activity was found to be positively related to constipation in patients undergoing chronic hemodialysis (Bossola et al., 2016). Involvement of the endogenous cannabinoid system in the regulation of gastrointestinal motility has been demonstrated *in vitro* and *in vivo* (Aviello et al., 2008). Lipopolysaccharide (LPS) was shown to reduce the amplitude and frequency of myoelectric spiking activity, and this was accompanied by a slowing of gastrointestinal transit (Li et al., 2010). Both cannabinoid-1 and cannabinoid-2 receptor antagonists were able to reverse the delayed intestinal transit induced by LPS (Li et al., 2010). However, evidence remains limited; therefore, further studies are needed to elucidate the exact mechanism.

### Colonic Propulsion

Several forms of contraction (mass movements, retrograde propulsion, segmentation movement) push contents through the colon. Propulsive contraction is designed to push and eventually discharge feces. The large contraction of colonic smooth muscle cells increases the intracavitary pressure, which are termed high-amplitude propagating contractions (HAPCs), the main propulsive contractile force which represent the main motor pattern related to mass movement (Vijayvargiya and Camilleri, 2019). Another motor pattern generated by enteric nerves is segmentation, which involves alternating contractions of the muscularis in a given region without forward propulsion of the luminal contents. The rhythmic slow-wave pattern naturally organizes the contractile activity of gastrointestinal muscles into phasic contractions (Sanders et al., 2014). The contents of the colon can also move in a retrograde direction, which are more pronounced after a meal, and this pattern of movement can potentially prevent rectal filling (Lin et al., 2017). The decreased frequency of HAPCs, increased low-amplitude propagated contractions, and reverse propulsive contraction frequency are related to the occurrence of constipation (Bassotti et al., 2003; Dinning and Di, 2011).

## Mechanism of Intestinal Electrolyte Transport

**FIGURE 3:**
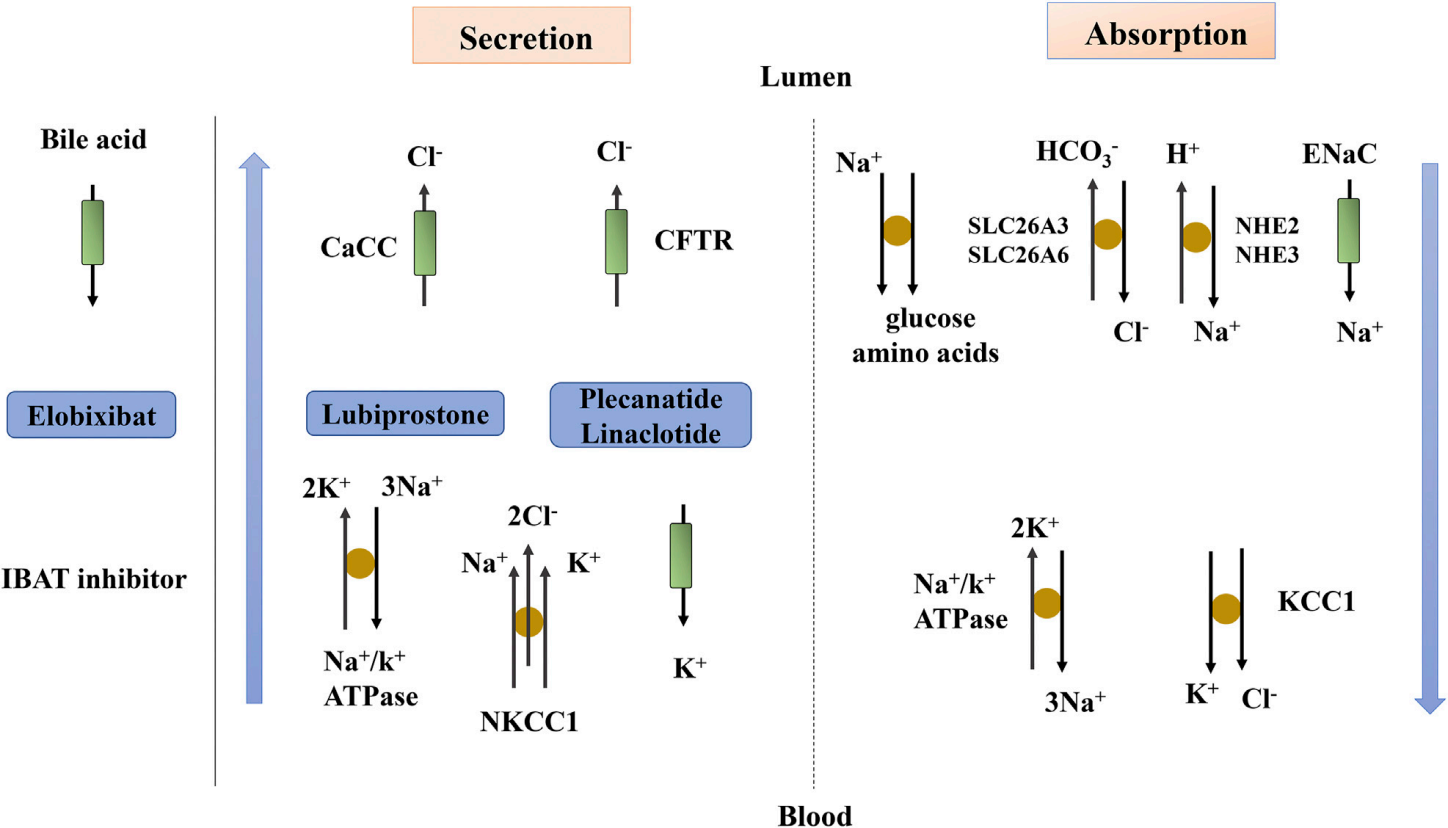
Fluid and electrolyte transport in the intestine. The blue arrow indicates the transport of fluid beside the cells. Lubiprostone, Plecanatide and Linaclotide act through ion transport. CaCC, Ca^2+^-activated Cl^−^ channel; CFTR, cystic fibrosis transmembrane conductance regulator; ENaC, epithelial sodium channel; IBAT, ileal bile acid transporter; KCC1, K^+^/Cl^−^ co-transporter; NHE, Na^+^/H^+^ exchanger; NKCC1, Na^+^/K^+^/Cl^−^ co-transporter; SLC26A3, Cl^−^ anion exchanger; SLC26A6, anion exchange transporter.

Electrolyte imbalance can lead to muscle weakness, thus accelerating the occurrence of chronic constipation. The modulation of ion channels and exchangers in epithelial cells can promote intestinal secretion, thereby enhancing gastrointestinal transit and promoting fecal excretion. This is also the mechanism through which some drugs exert their laxative effect (Figure 3). Under normal physiological conditions, many ion channels and exchangers exist in the intestinal epithelium, which play an important role in maintaining the balance of intestinal absorption and secretion (Kagnoff, 2014). The main mechanisms associated with ion fluxes are nutrient-coupled Na^+^ absorption, electroneutral NaCl absorption, electrogenic Na^+^ absorption, and Cl^−^ secretion (Kato and Romero, 2011).

### Mechanism of Intestinal Electrolyte Absorption

#### Nutrient-Coupled Na^+^ Absorption

Enteral nutrient-coupled Na^+^ absorption involves sodium-glucose transporters and Na-amino acid cotransporters, in which Cl^−^ and fluid are absorbed *via* a paracellular pathway (Kato and Romero, 2011). This also occurs in the presence of an adverse osmotic gradient (Wright and Loo, 2000).

#### Electroneutral NaCl Absorption

The coupled NaCl absorption mechanism occurs in both the small intestine and colon and is mediated by the coupling activity of the anion exchange transporter SLC26A6 or Cl^−^ anion exchanger SLC26A3 and the sodium/hydrogen exchanger family (NHE2 or NHE3) on the surface of epithelial cells (Patel-Chamberlin et al., 2016; Barrett, 2017). Na^+^ and Cl^−^ enter the cell cytosol through these transporters, and are then transported across the basolateral membrane *via* the Na^+^/K^+^-ATPase and K^+^/Cl^−^ cotransporters. Studies have shown that inhibiting SLC26A3, thus, blocking intestinal fluid absorption, can be used to effectively treat the main types of constipation (Haggie et al., 2018).

#### Electrogenic Na^+^ Absorption

Epithelial sodium channel (ENaC) is present on the superficial epithelial cells of the distal colon and rectum. Na^+^ absorption occurs on the luminal epithelial membrane through the ENaC and is compensated for by the Na^+^ output of basolateral Na^+^/K^+^-ATPase. This mechanism is important in the distal colon (Barrett, 2017). CAP1/Prss8, an *in vivo* regulator of colonic ENaC, can activate ENaC activity (Malsure et al., 2014) and is up-regulated by aldosterone.

### Mechanism of Intestinal Electrolyte Secretion

Chloride ion transport in epithelial cells is the main determinant of fluid secretion, and Cl^−^ is mainly secreted by crypt cells in the whole small intestine and colon. The main Cl^−^ channels involved in intestinal fluid secretion are CFTR and Ca^2+^-activated Cl^−^ channels (CaCC). CFTR is a chloride channel regulated by cAMP and cGMP, which plays a leading role in intestinal secretion (Kunzelmann et al., 2019). The increase of cAMP causes the apical membrane CFTR to open, leading to Cl^−^ outflow and intracellular depolarization (Dawson, 1991). Then, the increase in cAMP causes cAMP-dependent K^+^ channels in the basement membrane to open, resulting in K^+^ outflow and intracellular hyperpolarization to counteract the depolarization caused by the opening of apical membrane CFTR (Kunzelmann et al., 2001). The decreased intracellular Cl^−^ concentration induced by Cl^−^ outflow enhances the activity of the Na^+^/K^+^/2Cl^−^ co-transporter (NKCC1) in the basement membrane, transporting Cl^−^ into the cell through the basement membrane. Driven by Na^+^/K^+^-ATPase, NKCC1, and K^+^ channels, Cl^−^ enters the cell through the basement membrane, generating a Cl^−^ gradient across the epithelial cell top membrane. The activation of CaCC causes Cl^−^ outflow, and the secretion of Cl^−^ drives water transport (Jakab et al., 2013; Camilleri et al., 2017).

## Mechanism of Intestinal Fluid Transport

### Intestinal Fluid Transport Pathway

Colonic water absorption is the final link of intestinal water absorption in the body, and constipation is closely related to a disorder in the colonic fluid transport system. Intestinal epithelial fluid can be transported *via* paracellular and transcellular routes. Colonocytes are linked by tight junctions that impede fluid movement. This limits cell bypass absorption, and cross-cell transport becomes the main pathway of colonic fluid absorption (Sundell and Sundh, 2012). The transcellular route involves free diffusion, co-transport, and AQPs pathways (Laforenza et al., 2005). Among these, AQPs, which act as a special channel for the rapid transport of water molecules and small molecular solutes, play an important role in maintaining liquid homeostasis.

### Aquaporins in the Intestinal Tract

AQPs are a family of water channel molecules (AQP0-12) that promote the movement of water from areas of low permeability to areas of high permeability under the action of osmotic gradients (Sisto et al., 2019). AQPs are located on the cell membrane where they form channels to control the inflow and outflow of water. AQPs play a pivotal role in the regulation of intestinal absorption, secretion, and water metabolism by mediating the transmembrane transport of water molecules.

**FIGURE 4:**
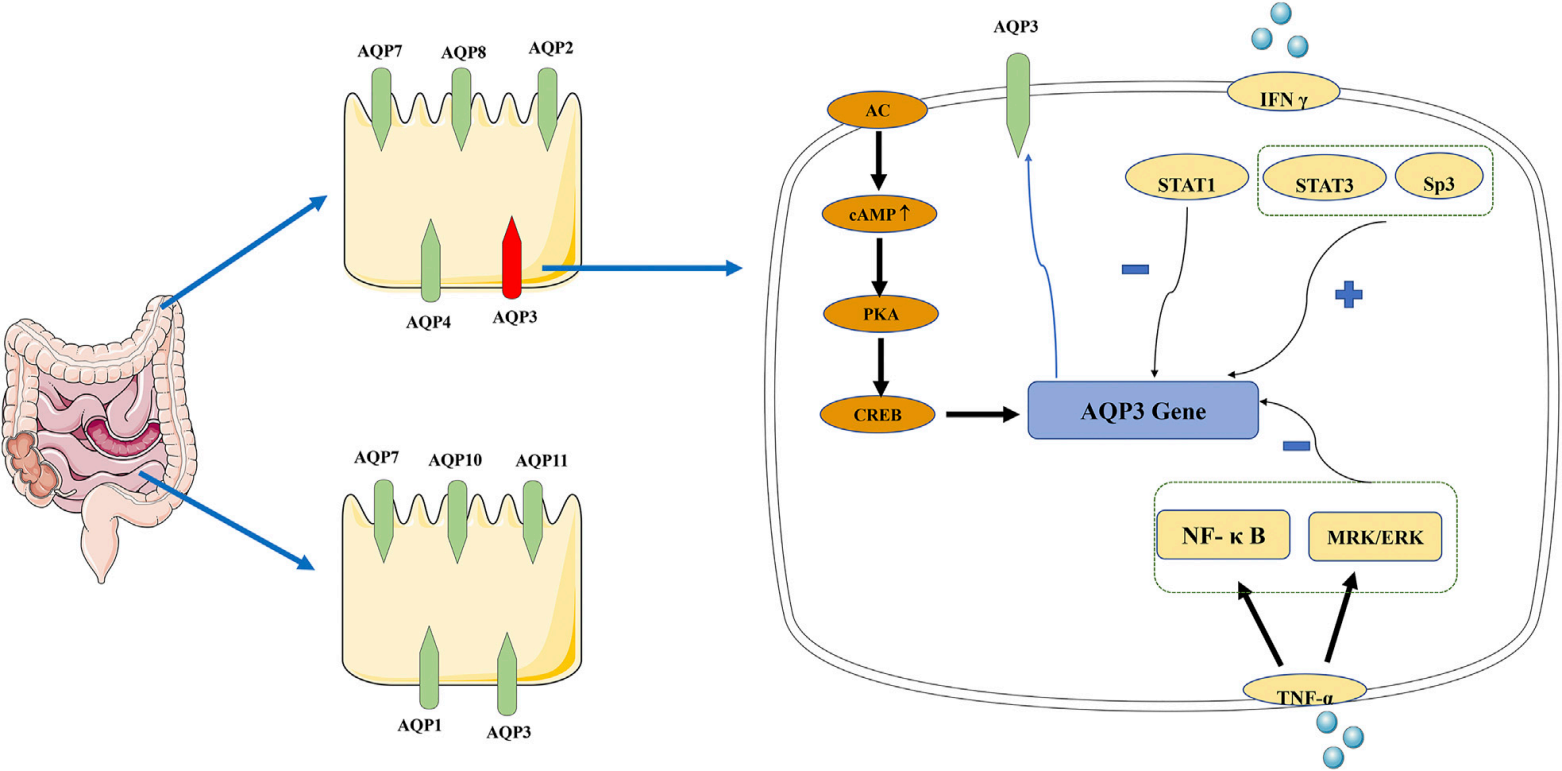
Distribution of AQPs in intestinal tract and signal transduction mechanism of AQP3. AQP1, AQP3, AQP7, AQP10, and AQP11 are highly expressed in the small intestine, while AQP2, AQP3, AQP4, AQP7, and AQP8 are the main subtypes in the colon. “+” indicate a promoting role, “−” indicate an inhibition role. In AQP3 expression, there are two pathways: AC-mediated short-time regulation and long-term regulation at the transcriptional level. AC, adenylate cyclase; AQP, aquaporin; cAMP, cyclic adenosine monophosphate; CREB, cAMP response element-binding protein; PKA, protein kinase A; TNF-α, tumor necrosis factor alpha.

Many studies have shown that AQP3 is a key subtype of colonic AQPs, and its expression level affects water transport in the intestinal tract. Up-regulation of AQP3 expression in colonic epithelial cells can lead to severe constipation (Zhu et al., 2017). AQPs can be regulated (Figure 4) through a change in their activity or number, which is also called short-term regulation. AQPs are targets of VIP that can change the content of AQPs on the cell membrane through the cellular protein kinase A (PKA) system to regulate the membrane permeability to water. VIP up-regulates the expression of AQP3 and its mRNA by activating the cAMP-dependent PKA pathway (Itoh et al., 2003), blocking the movement of Cl^−^ into the intestinal cavity and that of osmotic water from the cells to the cavity (Hamabata et al., 2002). PKA also phosphorylates cAMP response element-binding protein (CREB); the phosphorylated CREB then stimulates AQPs gene transcription (Ikeda and Matsuzaki, 2015).

The other mechanism refers to the increased AQPs synthesis, mRNA, and protein expression at the transcriptional level, which is termed long-term regulation. Studies have shown that AQPs promote intestinal function through mechanisms that may involve changes in signaling. NF-κB, a key signal in the long-term regulation of AQP3, down-regulates the expression of AQP3 (Zhan et al., 2020). Tumor necrosis factor alpha (TNF-α) reduces the expression of AQP3 in HT-29 cells through MRK/ERK and NF-κB signaling (Peplowski et al., 2017). The binding of Sp3 transcription factor to the AQP3 promoter can partially prevent the down-regulation of AQP3 expression induced by TNF-α. IFN-γ, a key factor of impaired epithelial transport and barrier function, can increase epithelial permeability by inhibiting the expression of AQP3. STAT1 has been shown to partially block the down-regulation of AQP3 expression induced by IFN-γ, while STAT3 and Sp3 can increase AQP3 expression (Peplowski et al., 2018).

A growing body of studies has investigated the importance of the gastrointestinal water transport system in intestinal function and the effect of AQPs on intestinal fluid secretion and chronic constipation. However, the mechanisms of AQPs action on intestinal fluid and constipation remain unclear. Further studies on the expression and function of AQPs in the intestinal tract and the mechanism of water transport should provide information for the development of new laxatives.

## Action Mechanism of Drugs for Treating Constipation

With a better understanding of the mechanisms of chronic constipation and continued advances in pharmaceutical development, an expanding array of treatment approaches have been developed. At present, drugs are the mainstay for patients with chronic constipation. Many studies have reported the efficacy and safety of laxatives in patients with constipation. Intervention with laxatives can alter the intestinal environment by affecting gut motility, ion transport, and liquid absorption/secretion, which are beneficial for patients with constipation. The main categories of approved drugs for the treatment of constipation are osmotic laxatives, stimulant laxatives, secretagogues, serotonergic agents, and ileal bile acid transporter inhibitors (Black and Ford, 2018). Drugs used for the treatment of chronic constipation are listed in Table 1. The goal of such medications is to promote fecal excretion and relieve constipation-related symptoms, and to improve the patients’ quality of life. However, different drugs act *via* different pathways, and a further understanding of their mechanisms, combined with that of ion transport and the expression of AQPs, may lead to the development of promising drugs.

**TABLE 1:** Summary of drugs for the treatment of constipation.

Category	Medication	Dosage	Possible side effects	Mechanism of action	Molecular signal
Bulking agents	Insoluble fiber	25–30 g/day	Bloating	Luminal water binding increases stool volume and soften stool	Not involved
	Soluble fiber				Not involved
Osmotic laxatives	Lactulose	15–25 ml/day	Bloating, abdominal pain	Produce the osmotic gradient in the cavity, increase the moisture in the cavity and soften the stool	Not involved
	Polyethylene glycol	10–20 g/day	Abdominal distention, diarrhea		Not involved
	MgSO_4_	5–20 g/day	Electrolyte disorder, diarrhea		Mg^2+^↑cAMP↑AQP3↑
Stimulant laxatives	Bisacodyl	5–10 mg/day	Abdominal cramps, diarrhea. Abdominal pain	Stimulate intestinal mucosa to secrete water and electrolytes and accelerate peristalsis	TNF-α↑PGE_2_↑AQP3↓
	Senna, rhubarb	No RCTs	Melanosis coli		PGE_2_↑ AQP3↓ AQP8↓
Secretagogues	Lubiprostone	24–72 μg/day	Nausea, vomiting, diarrhea	Increase intestinal fluid secretion and soften stool	Cl^−^↓ Na^+^↑ H_2_O↑
	Plecanatide	3 mg/day	Diarrhea		cGMP↑ Cl^−^↓ Na^+^↑ H_2_O↑
	Linaclotide	145 μg/day			
Serotonergic agents	Prucalopride	2 mg/day	Diarrhea, headache, nausea, abdominal pain	Selective action on 5-HT receptor	5-HT_4_↑ ICCs↑
IBAT inhibitor	Elobixibat	5–15 mg/day	Abdominal pain, diarrhea	Increase the concentration of bile acid in colon and promote fluid secretion	Bile acid↑ Cl^−^↓ H_2_O↑
Motilin receptor agonists	Mitemcinal (GM-611)	No RCTs	Not clear	Activation of motilin receptor and promote peristalsis	motilin↑
TGP	Paeonia	No RCTs	Not clear	Improve the function of ICCs and regulate neurotransmitters to accelerate peristalsis	ICCs↑ NO↓ NOS↓ VIP↓

5-HT, 5-hydroxytryptamine; AQP, aquaporin; cAMP, cyclic adenosine monophosphate; cGMP, cyclic guanosine monophosphate; IBAT, ileal bile acid transporter; ICCs, interstitial cells of Cajal; NO, nitric oxide; NOS, nitric oxide synthase; PGE_2_, prostaglandin E_2_; RCTs, randomized controlled trials; TGP, total glucosides of paeony; TNF-α, tumor necrosis factor alpha; VIP, vasoactive intestinal peptide.

### Bulking Agents

The American Gastroenterological Association recommends that use of a fiber supplement should be the initial treatment approach for constipation (Bharucha et al., 2013). Fiber is composed of high-molecular weight food components that cannot be degraded by intestinal enzymes thus it remains in the intestinal cavity and increases fecal volume. Insoluble fibers, such as wheat bran, may alter gut motility, thereby accelerating gastrointestinal transit and increasing the frequency of stools. Soluble fiber, such as psyllium, expands after absorbing water in the intestine, thus softening and increasing the volume of feces. Although fiber supplements are effective in the treatment of constipation, adverse reactions, such as bloating, are becoming a problem with long-term therapy (Wald et al., 2008).

### Osmotic Laxatives

If patients do not respond to fiber, then osmotic laxatives should be considered. Osmotic laxatives are poorly absorbed or non-absorbed substances, that produce intraluminal osmotic gradients, causing secretion of water and electrolytes into the lumen. This results in luminal water retention, an increase in stool water content and stool softening, thus facilitates stool passage (Krogh et al., 2017). These treatments are useful for patients with mild-to-moderate constipation, and the main side effects are diarrhea and abdominal distention.

#### Lactulose

As an osmotic laxative, lactulose has osmotic activity and can attract water to the colon cavity (Jouët et al., 2008). Since it is harmless to the human body and can effectively regulate the physiological rhythm of the colon, it is widely used to treat constipation in the elderly, pregnant women, and children. Adverse reactions are limited to the gastrointestinal system, with bloating and abdominal pain being the most common.

#### Polyethylene Glycol

Polyethylene glycol is a non-absorbable macromolecule belonging to the group of osmotic laxatives. Its mechanism of action is physical; it acts through local infiltration, retaining water in the colon cavity, thus softening feces, increasing fecal volume, and leading to unobstructed defecation. Polyethylene glycol can improve constipation-related symptoms (such as stool frequency and stool consistency) (Chassagne et al., 2017). Clinical studies have found that low-dose polyethylene glycol is significantly better than lactulose in improving constipation symptoms, with fewer adverse reactions (Attar et al., 1999). Polyethylene glycol can be used for the symptomatic treatment of constipation in children aged 6 months and older and in adults.

#### Poorly Absorbed Salts

Salt laxative MgSO_4_ can increase the intestinal osmotic pressure, prevent the absorption of water in the colon, increase the intestinal contents, and stimulate intestinal peristalsis, resulting in rapid and severe catharsis. The increased intracellular Mg^2+^ concentration activates adenylate cyclase (AC), which leads to an increase in cAMP. The increased cAMP concentration subsequently leads to the activation of PKA, which promotes CREB phosphorylation and AQP3 gene transcription (Ikarashi et al., 2011b). Excessive use of salt laxatives may induce electrolyte disorders and are therefore, not suitable for the elderly and patients with decreased kidney-function.

### Stimulant Laxatives

If the patient does not respond to osmotic laxatives, stimulant laxatives are recommended. Stimulant laxatives stimulate the intestinal mucosa and nerve plexus to secrete water and electrolytes, resulting in peristaltic contraction, thereby accelerating colonic transport. Stimulant laxatives are effective, and their side effects are known. Chronic use of stimulant laxatives does not seem to cause tolerance or rebound constipation. However, common side effects include diarrhea and abdominal pain (Wald, 2003).

#### Bisacodyl

Bisacodyl stimulates the secretion and motility of the small intestine and colon *via* the following mechanisms: increased secretion of TNF-α and prostaglandin E_2_ (PGE_2_) in the colon following the oral administration of bisacodyl. TNF-α and PGE_2_, as paracrine factors, act on the colonic mucosal epithelial cells resulting in an immediate reduction in AQP3 expression, thus exerting their laxative effects (Ikarashi et al., 2011a). In a 4-weeks trial, oral bisacodyl was reported to be safe and well-tolerated (Kamm et al., 2011). However, bisacodyl is associated with abdominal cramps and diarrhea.

### Secretagogues

Secretagogues are second-line drugs, the effects of which are similar to those of osmotic laxatives. Secretagogues act directly on intestinal epithelial cells, increasing fluid secretion into the intestinal cavity, thereby changing the consistency of stools and reducing the transit time in the colon (Luthra et al., 2019). However, these drugs are associated with side effects such as diarrhea when used clinically.

#### Lubiprostone

Lubiprostone is a prostaglandin E_1_ derivative, which can activate the intestinal chloride channel type 2 on the apical surface of small intestinal enterocytes, thereby reducing epithelial permeability and promoting intestinal fluid secretion. Lubiprostone selectively activates type 2 Cl^−^ channels in the parietal membrane of the gastrointestinal epithelium, resulting in the excretion of Cl^−^ ions through the periapical membrane. Additionally, sodium and water enter the intestinal cavity passively, increasing the secretion of fluid in the intestinal cavity (Gonzalez-Martinez et al., 2014). Lubiprostone can significantly increase stool frequency, improve stool consistency, and reduce straining, which makes it effective for the treatment of constipation (Nishii et al., 2020). The results of a meta-analysis showed that adverse reactions such as nausea, vomiting, and diarrhea were common (incidence rate, 2.4–75%) (Li et al., 2016). This may be related to the rapid flow of fluid into the small intestine after taking the medicine.

#### Guanylate Cyclase-C (GC-C) Receptor Agonists

Plecanatide and Linaclotide are GC-C receptor agonists that target GC-C receptors on the lumen of the intestinal epithelium, resulting in increased intestinal fluid secretion (Shah et al., 2018). By activating colonic epithelial GC-C receptors, the synthesis of intracellular and extracellular cGMP is increased (Busby et al., 2010). CFTR is activated indirectly by cGMP and induces epithelial cells to secrete Cl^−^ and HCO_3_^−^, inhibits Na^+^ absorption, and thus promotes intestinal water secretion (Layer and Stanghellini, 2014). Intestinal dilatation caused by increased intestinal fluid can promote intestinal movement, and therefore treat constipation. The reported efficacy of plecanatide and linaclotide is similar, and the most common side effect is diarrhea (Bassotti et al., 2018; Bassotti et al., 2019). Furthermore, increased cGMP can regulate abdominal pain (Silos-Santiago et al., 2013), thus, both drugs can relieve abdominal pain (Castro et al., 2013; Brenner et al., 2018).

### Serotonergic Agents

The use of osmotic and stimulant laxatives, either alone or in combination, may be considered in first-line drug therapy. Second-line agents, such as prucalopride are indicated in patients with an inadequate response or poor tolerance to a first-line drug. Studies have reported that exploiting epithelial targets with nonabsorbable serotonergic agents could provide safe and effective therapies. Serotonin agonists stimulate intestinal secretion and motility by activating 5-HT receptors in the gastrointestinal nervous system. Unlike other older non-selective 5-HT_4_ receptor agonists (e.g., cisapride and tegaserod), prucalopride is effective for the treatment of chronic constipation and has demonstrated an excellent safety profile (Hayat et al., 2017).

#### Prucalopride

Prucalopride is a high-affinity 5-HT_4_ receptor agonist with colonic prokinetic activity (Vijayvargiya and Camilleri, 2019). Prucalopride functions by activating 5-HT_4_ receptors in myenteric plexus neurons and stimulates HAPCs to increase colonic motility (Miner et al., 2016). This significantly increases intestinal muscle contraction, as well as stool frequency and consistency in patients with chronic constipation. Some studies have also found that prucalopride can increase the expression of c-kit mRNA in colonic tissue of rats with constipation, and then improve the function of ICCs, so as to promote colonic motility. It is effective at improving stool frequency, stool consistency and straining. The most common side effects include diarrhea, headache, nausea, and abdominal pain (Daniali et al., 2019). Multicenter, double-blind, randomized, placebo-controlled trials have demonstrated that prucalopride is superior to placebo in the short to medium term and can improve constipation in both men and women across a broad spectrum of ages and ethnicities (Camilleri et al., 2016). Nevertheless, this drug is considerably more expensive than conventional therapy. With knowledge that 5-HT_3_ receptors can participate in the activation of propulsive motility and secretory responses in the gut, 5-HT_3_ agonists have been developed and tested for the treatment of constipation (Mawe and Hoffman, 2013).

### Ileal Bile Acid Transporter Inhibitor

Bile acid can activate the secretory activity of colonic epithelial cells (Mekjian et al., 1971). Therefore, up-regulation of the colonic bile acid concentration can be used to treat patients with constipation. Bile acid, a natural laxative in the human body, has garnered attention for the treatment of chronic constipation because of its ability to promote colonic epithelial secretion (Keely et al., 2007). However, its efficacy and safety need to be further confirmed in large scale studies.

#### Elobixibat

Bile acid acts as a physiological laxative by activating AC, increasing mucosal permeability, and inhibiting apical Cl^−^/OH^−^ exchange to alter the transport of electrolytes and water in the lumen. Elobixibat can block the enterohepatic circulation of bile acid, up-regulate the synthesis of bile acid reaching the colon, and stimulate the secretion of fluid and electrolytes, thereby increasing fecal water content and gut motility (Mekjian et al., 1971). Increased gut motility facilitates stool passage. Few adverse reactions have been associated with this drug and they include abdominal pain and diarrhea. Abdominal pain with elobixibat may be related to its ability to induce dilatation and contraction (Taniguchi et al., 2018).

### Motilin Receptor Agonists

As a motilin receptor agonist, mitemcinal (GM-611) can stimulate and promote peristalsis of the gastrointestinal tract by acting on the motilin receptor (Sudo et al., 2007). This effect has been observed in animal models; however, due to a lack of clinical outcome data, the clinical significance of these studies has not been clearly demonstrated. Therefore, further clinical trials are required to confirm the efficacy and safety of mitemcinal in this population (Mozaffari et al., 2014).

### Probiotics

Probiotic consumption can regulate the intestinal microbiota of patients with constipation, which in turn, can improve gut motility. Some studies have shown that probiotics can be helpful for treating patients with constipation (improved stool frequency and stool consistency) with very few side effects (Ohkusa et al., 2019; Zhang et al., 2020). These studies have mainly involved the *bacteroides*, bifidobacterial, and lactobacilli. Recently, the positive impacts of SCFAs on gut motility and constipation were established (Chu et al., 2019). Nevertheless, since the effects of probiotics may be strain-specific and the exact mechanism of action remains unknown, more studies and randomized controlled trials are needed to confirm the effects of probiotics in patients with constipation (Dimidi et al., 2020).

### Traditional Chinese Medicine

Traditional Chinese Medicine (TCM) has a role in promoting gastrointestinal motility and has been used to treat constipation for more than 1,000 years in China. In recent years, there have been many reports about TCM in the treatment of constipation and the improvement of gastrointestinal function (Cirillo and Capasso, 2015). Therefore, TCM has garnered increasing attention as a promising alternative treatment for constipation. However, few studies have investigated its therapeutic mechanisms. Thus, the long-term efficacy and side-effect profiles of these medicines in modern medicine need to be determined. Further studies are needed to determine the exact mechanism for the observed recovery of intestinal function. Importantly, the composition of TCM is complex.

Senna and rhubarb are anthraquinone laxatives used widely in the treatment of intestinal constipation. Sennoside A exerts a laxative effect through its main bioactive component. Rheinanthrone, the active metabolite of sennoside A, can increase the production of PGE_2_ (Kon et al., 2014), thus participating in the regulation of intestinal peristaltic reflex. Emodin in rhubarb may also regulate water transport and absorption *via* the cAMP-dependent PKA/p-CREB signal pathway to change AQP3 (Zheng et al., 2014). Owing to its toxicity to the kidney and liver, we suggest that special attention should be paid to patients with kidney and liver diseases when using Senna drugs for a long period (Cao et al., 2018).

Total glucosides of paeony (TGP) are extracted from the root of Paeonia Lactiflora Pall. Studies have shown that TGP can improve the function of ICCs, block inhibitory neurotransmitters such as NO, NOS, and VIP, and increase the fecal volume and water content, as well as intestinal transit rate (Zhu et al., 2016).

## Conclusion and Prospect

As a common disease that seriously impacts the quality of life and mental health of patients, chronic constipation has attracted widespread attention. In general, impaired gut motility, fluid secretion/absorption, and electrolyte transport can result in decreased intestinal transit, reduced fluid secretion, and increased fluid reabsorption, which will eventually lead to chronic constipation. Further studies on the abnormal changes of the ENS, ANS, CNS, endocrine signaling, and microbiota would aid our understanding of constipation from the perspective of gut motility. Intestinal fluid and electrolyte transport are also strongly correlated with chronic constipation. As a subject for future research, ion channels and AQPs play critical roles in the transport of fluid. At present, studies on ion transport and AQPs in constipation are limited, and many complex mechanisms have not been clarified. Further experiments are warranted to demonstrate this mechanism.

With extended symptom duration, severity, and frustration, the occurrence of additional symptoms will also increase. Therefore, patients with chronic constipation usually require active treatment. The choice of therapeutic drugs should focus on the effectiveness of relieving the symptoms of constipation, the improvement of the intestinal environment, and the effectiveness and safety of long-term use. Preferentially, fiber/osmotic/stimulant laxatives should be considered. If these measures fail, prescription laxatives with different mechanisms of action may be used. Modifying the gut luminal environment through gut motility, fluid, and electrolytes will affect transit and secretion in the gut, thereby benefiting patients with chronic constipation. Intestinal fluid transport mediated by ion channels and AQPs is the key mechanism through which many laxatives exert their effects. An in-depth understanding of ion channels and water channels in constipation will provide a scientific basis for the development of synergistic and/or antagonistic drugs targeting specific channels. Nevertheless, further studies are still required to resolve the problem. Recently, the action of probiotics on gut motility was shown to be beneficial for constipation. However, the positive effects of probiotics depend on the specific probiotics used and the level applied. Therefore, the use of probiotics in the treatment of chronic constipation is promising and further studies are required. We hope that a better understanding of the pathogenesis of constipation and the mechanism of drug action may create new targets for the treatment of diseases that remain a major scourge worldwide.

## Glossary

5-HT: 5-hydroxytryptamine
AC: adenylate cyclase
Ach: acetylcholine
ANS: autonomic nervous system
AQPs: aquaporins
ATP: adenosine triphosphate
CaCC: Ca^2+^ activated Cl^−^ channel
cAMP: cyclic adenosine monophosphate
CFTR: cystic fibrosis transmembrane conductance regulator
cGMP: cyclic guanosine monophosphate
CGRP: calcitonin gene-related peptide
CNS: central nervous system
CREB: cAMP response element-binding protein
ECs: enterochromaffin cells
EGCs: enteric glial cells
ENaC: epithelial sodium channel
ENS: enteric nervous system
ExPANs: extrinsic primary afferent neurons
GC-C: guanylate cyclase-C
H_2_S: hydrogen sulfide
HAPCs: high-amplitude propagating contractions
ICCs: interstitial cells of Cajal
LPS: lipopolysaccharide
NKCC1: Na^+^/K^+^/2Cl^−^ co-transporter
NO: nitric oxide
NOS: nitric oxide synthase
PDGFRα^+^: platelet-derived growth factor receptor α-positive
PGE_2_: prostaglandin E_2_
PKA: protein kinase A
SCFAs: short-chain fatty acids
SP: substance P
TCM: Traditional Chinese medicine
TGP: Total glucosides of paeony
TNF-α: tumor necrosis factor alpha
VIP: vasoactive intestinal peptide
